# Relationship between intra‐abdominal hypertension, outcome and the revised Atlanta and determinant‐based classifications in acute pancreatitis

**DOI:** 10.1002/bjs5.29

**Published:** 2018-03-15

**Authors:** P. Marcos‐Neira, F. Zubia‐Olaskoaga, S. López‐Cuenca, L. Bordejé‐Laguna, on behalf of the Epidemiology of Acute Pancreatitis in Intensive Care Medicine study group, L. Bordejé, P. Arribas, E. Labarca, C. Lorencio, C. Fernández‐González, P. Araujo, V. González‐Sanz, E. Salgado, M. Duro, I. Navarrete, M. Sevilla, I. Alcalde, M. A. García‐García, J. M. Gutiérrez‐Rubio, M. del Carmen Córdoba, L. del Baño, A. Renedo, R. Carrasco, A. Menéndez, L. J. Yuste, A. Fernández‐Trujillo, D. Gutiérrez, A. Marqués, A. Guglieri, D. Pérez‐Martínez, L. Lage, M. Pilar Gracia‐Arnillas, R. Díaz‐Abad, M. A. Blasco, M. Mourelo, J. Iglesias, M. J. Pérez‐San José, E. García‐Mañosa, C. Velilla, J. H. de Gea, K. García‐Castillo, B. Martínez‐Palacios, A. Ortega, I. Fernández‐Simón, S. Rodríguez‐Ramos, M. Álvarez, R. Ramírez, A. Jordá, F. Martínez‐Lozano, N. Franco, C. Campos, D. F. López‐Hormiga, M. Loinaz, A. Caballero, S. M. Cortez, A. Margarit, M. J. Broch, J. Bonastre, A. M. Prieto de Lamo, J. Sánchez‐Ballesteros, F. Arbol, G. Leoz, M. Arroyo, M. Martínez‐Barrios, M. V. de la Torre, P. Nuevo, F. Minaya, T. Recio, J. Ignacio, M. Planella

**Affiliations:** ^1^ Department of Intensive Care Germans Trias i Pujol University Hospital Barcelona Spain; ^2^ Department of Intensive Care Donostia – San Sebastián University Hospital Guipúzcoa Spain; ^3^ Department of Intensive Care Getafe University Hospital Madrid Spain

## Abstract

**Background:**

The aim of this study was to analyse the relationship between intra‐abdominal hypertension (IAH) and severity of acute pancreatitis (AP) measured by the revised Atlanta classification (RAC) and determinant‐based classification (DBC). Secondary objectives were to assess IAH as a predictor of morbidity and mortality in the ICU.

**Methods:**

This prospective international observational study included patients admitted to the ICU with AP and at least one organ failure. Information was collected on demographics, severity scores at admission using RAC and DBC, organ failure, mechanical ventilation, continuous renal replacement therapy (CRRT), surgery and mortality. Maximum intra‐abdominal pressure (IAP) during ICU stay was used for analysis.

**Results:**

Some 374 patients were included. The hospital mortality rate was 28·9 per cent. IAP was measured in 301 patients (80·5 per cent), of whom 274 (91·0 per cent) had IAH and 103 (34·2 per cent) acute compartment syndrome. A higher IAH grade was more likely in patients with severe AP (42 per cent for grade I versus 84 per cent for grade IV) and acute critical pancreatitis (9 versus 25 per cent; P = 0·001). Compared with grade I IAH, patients with grade IV had more infected necrosis (16 versus 28 per cent; P = 0·005), need for surgery (27 versus 50 per cent; P = 0·006), mechanical ventilation (53 versus 84 per cent; P = 0·007) and requirement for CRRT (22 versus 66 per cent; P < 0·001). IAH predicted shock (area under receiver operating characteristic (ROC) curve (AUC) 0·79, 95 per cent c.i. 0·73 to 0·84), respiratory failure (AUC 0·82, 0·77 to 0·87), renal failure (AUC 0·93, 0·89 to 0·96) and mortality (AUC 0·89, 0·86 to 0·93).

**Conclusion:**

IAH was associated with severity of AP classified according to both RAC and DBC systems. IAP grade can predict outcome of AP during ICU stay.

## Introduction

**Figure 1:**
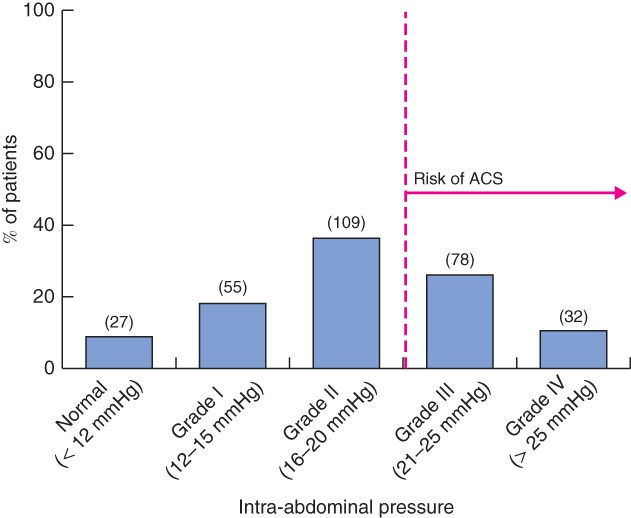
Classification and distribution of intra‐abdominal hypertension in the Epidemiology of Acute Pancreatitis in Intensive Care Medicine (EPAMI) study. Values in parentheses are numbers of patients. ACS, acute compartment syndrome

Intra‐abdominal hypertension (IAH) is defined as a repeated pathological intra‐abdominal pressure (IAP) increase of 12 mmHg or more. When IAP is above 20 mmHg (with or without an abdominal perfusion pressure below 60 mmHg) and associated with organ failure (mainly cardiovascular, respiratory or renal dysfunction), it is called abdominal compartment syndrome (ACS). According to its severity, IAH is classified in four categories^1^, ^2^, ^3^ (*Fig*. 1).

IAH and ACS are common events in acute pancreatitis (AP), affecting up to 60–80 per cent of patients in some studies^4^, ^5^. The proportion of patients thought to have IAH depends on where the threshold is set. With an IAP greater than 15 mmHg, over 75 per cent of patients might be classified as having IAH^4^, ^5^, often within the first 12 h of their apparent illness^6^, ^7^. Some patients with AP may develop ACS (25–56 per cent)^4^, ^5^, ^6^, ^7^, ^8^, associated with a high mortality rate (50–75 per cent)^4^, ^5^, ^6^, ^7^, ^8^. IAH in AP with organ failure is generally thought to reflect visceral oedema due to the severity of the inflammatory process, potentially compounded by aggressive fluid resuscitation (volume overload).

Recently, two classification systems have been developed for patients with AP to predict mortality risk: the revised Atlanta classification (RAC)^9^ and the determinant‐based classification (DBC)^10^. These two systems are useful to characterize case mix and to compare the effectiveness of treatments between centres. Initial validation studies^11^, ^12^ showed that the two classifications were equally accurate in predicting mortality, whereas other systems used to assist clinicians in the evolution of AP during an ICU stay have low accuracy^13^, ^14^. IAH may be useful to predict local pancreatic and systemic complications^8^, ^15^, ^16^, ^17^, ^18^.

This study sought to examine the relationship between IAP and the RAC and DBC systems. Secondary objectives were to assess morbidity and mortality associated with IAH in patients with AP.

## Methods

This was a prospective observational multicentre international cohort study conducted over a 1‐year period. Following the consensus conference on the management of AP under the auspices of the Spanish Intensive Care Society (SEMICYUC)^3^, invitations were sent to all international ICUs that contributed to the Epidemiology of Acute Pancreatitis in Intensive Care Medicine (EPAMI) study. In addition, several ICUs in South America were invited to participate. Participating centres are shown in Table S1 (supporting information).

Study inclusion criteria were: patient aged over 18 years, admitted to an ICU with a diagnosis of AP and at least a single organ failure. The following definitions were used: AP diagnosed by at least two of the following three criteria – upper abdominal pain, amylase or lipase levels raised at least threefold with respect to laboratory upper limits of normal, appropriate imaging findings; organ failure – RAC and DBC definitions, adapted to intensive care, using SEMICYUC consensus conference criteria^3^; shock – systolic arterial pressure below 90 mmHg or a reduction of 40 mmHg in basal systolic arterial pressure, with tissue hypoperfusion signs where lactate concentration was greater than 3 mmol/l and central venous oxygen saturation was less than 70 per cent^19^; respiratory failure – either basal arterial partial pressure of oxygen (Pao_2_) below 60 mmHg, or Pao_2_ or fraction of inspired oxygen (Fio_2_) of less than 300 mmHg with supplementary oxygen; acute renal failure – increase in serum creatinine to more than twice the upper limit of normal and/or less than 0·5 ml per kg per h for more than 12 h according to urinary output criteria for the Acute Kidney Injury Network (AKIN) classification; IAH – a repeated pathological IAP increase of 12 mmHg or above; and ACS – when IAP was above 20 mmHg with or without an abdominal perfusion pressure below 60 mmHg and associated with an organ failure that was cardiovascular, respiratory or renal^1^, ^20^.

There were no exclusion criteria.

Study recruitment was from 1 January to 31 December 2013. The study was approved by Donostia – San Sebastián University Hospital ethics committees and endorsed at other centres. Each patient provided signed informed consent before entering the study. All data were anonymized.

Data were collected for all patients relating to: age, sex, aetiology, shock, respiratory failure, acute renal failure, local complications, two widely used ICU scores to predict mortality (Acute Physiology And Chronic Health Evaluation (APACHE) II and Sequential Organ Failure Assessment (SOFA) in first 24 h), use of mechanical ventilation, continuous renal replacement therapy (CRRT), surgery, ICU length of stay (LOS), overall hospital LOS and hospital mortality.

IAP was measured via the urinary bladder at end expiration in the completely supine position after ensuring that abdominal muscle contractions were absent and with the transducer zeroed at the level of the mid‐axillary line, according to the updated consensus definition from the World Society of the Abdominal Compartment Syndrome^1^, ^20^. IAP was measured every 6 h daily, and maximum IAP value obtained during the ICU stay was the one analysed. Owing to design issues, the day on which value occurred was not collected. Maximum IAP was graded according to the same consensus (Fig. 1).

### Statistical analysis

Quantitative variables with a normal distribution were expressed as mean(s.d.) and those with a non‐parametric distribution as median (i.q.r.) values. Categorical data were expressed as frequencies and percentages. The χ^2^ test was used to compare categorical data and proportions, and Student's t test or the Mann–Whitney U test, as appropriate, to compare continuous variables. Receiver operating characteristic (ROC) curve analysis was used to diagnose the ability of IAP to predict organ failure and mortality. An α level of 0·05 was used to determine statistical significance. Patients with missing data were not analysed. All data were analysed using IBM SPSS® version 22.0 (IBM, Armonk, New York, USA).

## Results

A total of 405 patients diagnosed with AP and at least one organ failure were admitted to the ICU. Thirty‐one patients (7·7 per cent) did not provide informed consent, so a total of 374 patients were included from 46 hospitals. The majority of patients were men (62·6 per cent) and mean age was 60·4(15·6) years. The most frequent aetiology was biliary pathology affecting 174 patients (46·5 per cent). Patients were critically ill as indicated by mean APACHE II (16·1(8·2)) and SOFA (6·6(4·5)) scores. The overall hospital mortality rate was 28·9 per cent. Some 229 patients (61·2 per cent) developed shock, 234 patients (62·6 per cent) developed respiratory failure and 235 (62·8 per cent) had renal failure. Half of the patients (52·1 per cent) required ventilatory support, 109 (29·1 per cent) renal support and 110 (29·4 per cent) needed surgery (including patients who needed decompressive laparotomy). Of 108 patients who died, 38 (35·2 per cent) died at an early stage because of a systemic inflammatory response syndrome (SIRS) and 23 (21·3 per cent) as a result of pancreatic infection. Median ICU LOS was 7 days and median hospital LOS was 24 days. Baseline characteristics and outcomes are summarized in *Table* 1.

**Table 1:** Patient characteristics

	No. of patients (*n* = 374)*
No. of centres	46
No. of patients per centre†	8·1(4·5)
No. of patients with IAP measured	301 (80·5)
Age (years)†	60·4(15·6)
Sex ratio (M : F)	234 : 140
Aetiology of AP	
Biliary	174 (46·5)
Alcoholic	82 (21·9)
Idiopathic	62 (16·6)
Other	56 (15·0)
APACHE II score in first 24 h†	16·1(8·2)
SOFA in first 24 h†	6·6(4·5)
Shock	229 (61·2)
Persistent	148 of 229 (64·6)
Renal failure	235 (62·8)
Persistent	141 of 235 (60·0)
Respiratory failure	234 (62·6)
Persistent	164 of 234 (70·1)
Mechanical ventilation	195 (52·1)
CRRT	109 (29·1)
Surgery	110 (29·4)
ACS	103 of 301 (34·2)
Decompressive laparotomy	9 of 301 (3·0)
ICU LOS‡	7 (4–21)
Hospital LOS‡	24 (14–41)
Overall mortality	108 (28·9)
Cause of death	
Initial SIRS	38 of 108 (35·2)
Pancreatic infection	23 of 108 (21·3)
Non‐pancreatic infection	14 of 108 (13·0)
Other	33 of 108 (30·6)

* With percentages in parentheses unless indicated otherwise; values are †mean(s.d.) and ‡median (i.q.r.). IAP, intra‐abdominal pressure; AP, acute pancreatitis; APACHE, Acute Physiology And Chronic Health Evaluation; SOFA, Sequential Organ Failure Assessment; CRRT, continuous renal replacement therapy; ACS, acute compartment syndrome; LOS, length of stay; SIRS, systemic inflammatory response syndrome.

IAP was measured in 301 patients (80·5 per cent). Mean maximum IAP was 19·2(5·8) mmHg. Of these patients, 274 (91·0 per cent) developed IAH, among whom 110 (36·5 per cent) were considered at risk of developing ACS because they had an IAP above 20 mmHg. ACS developed in 103 patients (34·2 per cent), of whom nine (8·7 per cent) underwent decompressive laparotomy; three had an IAP of 21–25 mmHg and six an IAP above 25 mmHg. Seven of these nine patients died.

### Relationship between graded intra‐abdominal hypertension and clinical evolution

**Figure 2:**
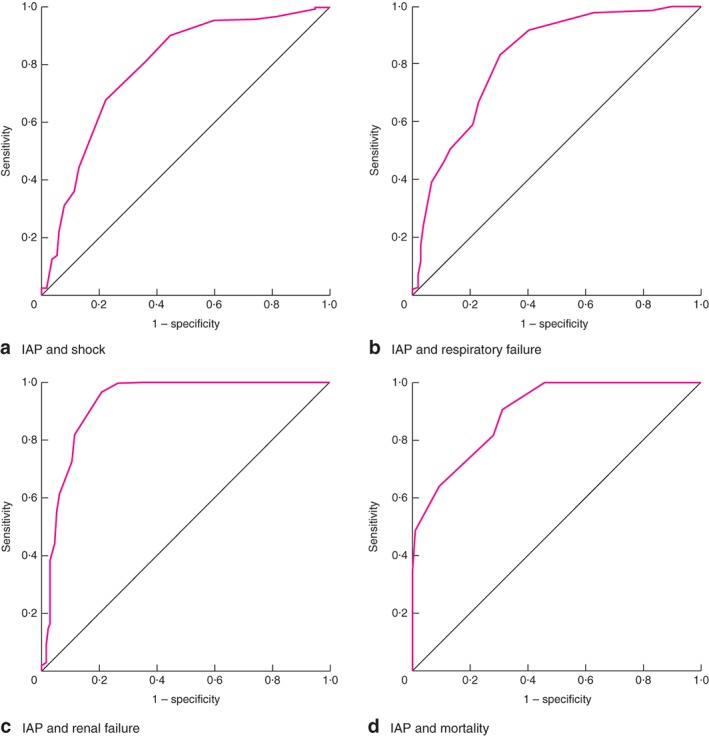
Receiver operating characteristic (ROC) curve analysis for intra‐abdominal pressure (IAP) and **a** shock, **b** respiratory failure, **c** renal failure and **d** mortality

There was a direct significant relationship between graded IAH and the development of shock (*P* < 0·001), respiratory failure (*P* = 0·007), renal failure (*P* < 0·001), need for mechanical ventilation (*P* = 0·007) and CRRT (*P* < 0·001) (*Table* 2). Using ROC curves, the area under the curve (AUC) of IAP to predict shock was 0·79 (95 per cent c.i. 0·73 to 0·84), to predict respiratory failure 0·82 (0·77 to 0·87), to predict renal failure 0·93 (0·89 to 0·96) and to predict mortality 0·89 (0·86 to 0·93) (all *P* < 0·001) (*Fig*. 2). ROC analysis showed that the best cut‐off point to predict shock was an IAP of 15·5 mmHg (sensitivity 89·9 per cent, specificity 55·8 per cent, positive predictive value (PPV) 77·2 per cent, negative predictive value (NPV) 76·8 per cent), to predict respiratory failure an IAP of 17·5 mmHg (sensitivity 82·7 per cent, specificity 70·1 per cent, PPV 82·7 per cent, NPV 70·1 per cent), to predict renal failure an IAP of 18·5 mmHg (sensitivity 81·5 per cent, specificity 88·7 per cent, PPV 90·1 per cent, NPV 79·2 per cent) and to predict mortality in the ICU an IAP of 19·5 mmHg (sensitivity 81·4 per cent, specificity 72·1 per cent, PPV 59·1 per cent, NPV 89·1 per cent).

**Table 2:** Intra‐abdominal pressure classification and outcome in 301 patients

	IAP					
	Normal (*n* =27)	Grade I (*n* = 55)	Grade II (*n* = 109)	Grade III (*n* = 78)	Grade IV (*n* = 32)	*P* †
Shock	7 (26)	33 (60)	75 (69·8)	56 (72)	24 (75)	< 0·001
Renal failure	15 (56)	33 (60)	66 (60·6)	60 (77)	27 (84)	< 0·001
CRRT	0 (0)	12 (22)	31 (28·4)	34 (44)	21 (66)	< 0·001
Respiratory failure	10 (37)	32 (58)	76 (69·7)	55 (71)	28 (88)	0·007
Mechanical ventilation	5 (19)	29 (53)	62 (56·9)	53 (68)	27 (84)	0·007
Infected necrosis	3 (11)	9 (16)	30 (27·5)	32 (41)	9 (28)	0·005
Surgery	5 (19)	15 (27)	28 (25·7)	35 (45)	16 (50)	0·006
Decompressive laparotomy	0 (0)	0 (0)	0 (0)	3 (4)	6 (19)	
Nutrition						0·009
None	–	9 (16)	32 (29·4)	16 (21)	6 (19)	
Enteral	–	22 (40)	26 (23·9)	15 (19)	6 (19)	
Parenteral	–	23 (42)	45 (41·3)	43 (55)	19 (59)	
Enteral and parenteral	–	1 (2)	6 (5·5)	4 (5)	1 (3)	
ICU LOS (days)*	5 (3–7)	6 (3–14·3)	10 (5–27)	18 (6·8–32·3)	11 (3·5–25)	< 0·001‡
Hospital LOS (days)*	15 (12–26)	22 (13–38)	24 (14–43)	30 (8–59)	17 (8–29)	< 0·001‡
Mortality	1 (4)	11 (20)	33 (30·3)	31 (40)	21 (66)	< 0·001
Cause of death						< 0·001
Initial SIRS	0 (0)	5 of 11 (45)	13 of 33 (39)	7 of 31 (23)	7 of 21 (33)	
Pancreatic infection	0 (0)	2 of 11 (18)	4 of 33 (12)	9 of 31 (29)	7 of 21 (33)	
Non‐pancreatic infection	0 (0)	1 of 11 (9)	7 of 33 (21)	3 of 31 (10)	2 of 21 (10)	
Other	1 of 1 (100)	3 of 11 (27)	9 of 33 (27)	12 of 31 (39)	5 of 21 (24)	

Values in parentheses are percentages unless indicated otherwise;

* values are median (i.q.r.). CRRT, continuous renal replacement therapy; LOS, length of stay; SIRS, systemic inflammatory response syndrome.

† χ^2^ test, except ‡Mann–Whitney *U* test.

There was a direct significant relationship between the development of infected pancreatic necrosis, need for surgery and graded IAH (*P* = 0·006). A direct relationship existed between ICU and hospital LOS and graded IAH (*P* < 0·001). Enteral nutrition was used most frequently in patients with grade I (40 per cent) and II (23·9 per cent) IAP than in those in grade III or IV (both 19 per cent) (*P* = 0·009) (*Table* 2).

### Relationship between graded intra‐abdominal hypertension, revised Atlanta and determinant‐based classifications

When RAC was applied, there were 163 patients (43·6 per cent) with moderate and 211 (56·4 per cent) with severe AP. With the DBC, there were 135 patients (36·1 per cent) with moderate forms of AP, 175 (46·8 per cent) with severe AP and 64 (17·1 per cent) with critical AP. Clinicians measured IAP more frequently in severe and critical forms of AP than in moderate ones (*P* < 0·001). There was a direct relationship between graded IAH and the severity of AP according to the RAC and DBC systems (*P* = 0·001). For example, there were no critical forms of AP according to the DBC in patients with a normal IAP, and 74 per cent of patients with grade III IAH and 84 per cent of patients with grade IV IAH developed a severe form of AP according to the RAC (*Table* 3).

**Table 3:** Severity of acute pancreatitis and intra‐abdominal hypertension in the 374 patients according to revised Atlanta and determinant‐based classifications

	Revised Atlanta classification		Determinant‐based classification		
	AMP (*n* = 163)	ASP (*n* = 211)	AMP (*n* = 135)	ASP (*n* = 175)	ACP (*n* = 64)
No. with acute AP and measurement of IAP (*n* = 301)	116 (71·2)	185 (87·7)†	94 (69·6)	146 (83·4)†	61 (95·3)†
IAP					
Normal (*n* = 27)	24 (89)	3 (11)*	20 (74)	7 (26)*	0 (0)*
Grade I (*n* = 55)	32 (58)	23 (42)*	28 (51)	22 (40)*	5 (9)*
Grade II (*n* = 109)	35 (32·1)	74 (67·9)*	28 (25·7)	58 (53·2)*	23 (21·1)*
Grade III (*n* = 78)	20 (26)	58 (74)*	13 (17)	40 (51)*	25 (32)*
Grade IV (*n* = 32)	5 (16)	27 (84)*	5 (16)	19 (59)*	8 (25)*

Values in parentheses are percentages. AMP, acute moderate pancreatitis; ASP, acute severe pancreatitis; ACP, acute critical pancreatitis; AP, acute pancreatitis; IAP, intra‐abdominal pressure.

* *P* = 0·001,

† *P* < 0·001 *versus* AMP (χ^2^ test).

## Discussion

This study has shown a significant relationship between graded IAH and the severity of AP stratified by the RAC and DBC systems. IAP should be measured in patients with AP as it can predict severity and potentially influence management. The study confirmed the relationships between IAP, organ failure and mortality in the ICU in line with other observations^6^, ^16^, ^17^, ^18^. Although one study^17^ found that an IAP of 9 mmHg provided the best predictive value of 30‐day mortality (sensitivity 86 per cent, specificity 87 per cent; AUC ROC 0·91), this very low cut‐off point probably reflects the fact that patients in the present study all had AP and at least one organ failure.

Just over one‐third of patients in the present study developed ACS, similar to other results ranging from 27 to 45 per cent^15^, ^18^, ^21^. Unlike earlier studies where rates of decompressive laparotomy were high (74–77 per cent)^18^, ^21^, only 8·7 per cent of the present cohort diagnosed with ACS were treated in this way. Some patients in the present series were treated with effective medical decompressive methods (nasogastric and rectal tubes, percutaneous drainage of abdominal collections, negative fluid balance with diuretics and renal support), as recommended elsewhere^1^, ^2^, ^3^. As the treatment of ACS was not specified by protocol in the study design, participating centres may not have considered that existing data from decompressive laparotomy justified this procedure. In both of the above studies^18^, ^21^, the mortality rate of patients with ACS was high (49 and 54·5 per cent respectively). The mortality rate was also high in the present study: 40 per cent for patients with grade III IAH, 66 per cent for patients with grade IV IAH, and seven of nine patients (78 per cent) who had decompressive laparotomy.

This study had limitations. EPAMI was an observational study and its purpose was focused on epidemiology and AP classification. IAP and IAH were analysed as secondary variables. IAP was not measured in all patients, and the timing of the highest IAP may not have been recorded. Despite these shortcomings, IAP was significantly associated with severity of AP according to both the RAC and DBC.

Graded IAP was predictive of outcome in patients with AP during the ICU stay in the present study. Its true value in predicting the development of organ failure and mortality should be investigated in a study designed specifically for this purpose.

## Collaborators

Other members of the EPAMI study group: L. Bordejé, P. Arribas, E. Labarca, C. Lorencio, C. Fernández‐González, P. Araujo, V. González‐Sanz, E. Salgado, M. Duro, I. Navarrete, M. Sevilla, I. Alcalde, M. A. García‐García, J. M. Gutiérrez‐Rubio, M. del Carmen Córdoba, L. del Baño, A. Renedo, R. Carrasco, A. Menéndez, L. J. Yuste, A. Fernández‐Trujillo, D. Gutiérrez, A. Marqués, A. Guglieri, D. Pérez‐Martínez, L. Lage, M. Pilar Gracia‐Arnillas, R. Díaz‐Abad, M. A. Blasco, M. Mourelo, J. Iglesias, M. J. Pérez‐San José, E. García‐Mañosa, C. Velilla, J. H. de Gea, K. García‐Castillo, B. Martínez‐Palacios, A. Ortega, I. Fernández‐Simón, S. Rodríguez‐Ramos, M. Álvarez, R. Ramírez, A. Jordá, F. Martínez‐Lozano, N. Franco, C. Campos, D. F. López‐Hormiga, M. Loinaz, A. Caballero, S. M. Cortez, A. Margarit, M. J. Broch, J. Bonastre, A. M. Prieto de Lamo, J. Sánchez‐Ballesteros, F. Arbol, G. Leoz, M. Arroyo, M. Martínez‐Barrios, M. V. de la Torre, P. Nuevo, F. Minaya, T. Recio, J. Ignacio, M. Planella.

## Disclosure

The authors declare no conflict of interest.

**Supporting information**

Additional supporting information can be found online in the supporting information tab for this article.

## Supporting information


**Table S1** Centres in the EPAMI study
