# Supplementary material for: Relationship between intra‐abdominal hypertension, outcome and the revised Atlanta and determinant‐based classifications in acute pancreatitis
Source: BJS Open. 2018 Mar 15;1(6):175–81. doi: 10.1002/bjs5.29 (PMC5989946; doi:10.1002/bjs5.29)
Supplement: Supplementary file 1 — Table S1 Centres in the EPAMI study [file BJS5-1-175-s001.docx]

Supporting Information (ST1).

Centres in EPAMI study

| **Centre** | **Number of patients** | **Centre** | **Number of patients** |
| --- | --- | --- | --- |
| San Cecilio University Hospital (Granada) | 23 | La Ribera University Hospital (Alzira) | 7 |
| Germans Trias i Pujol University Hospital (Badalona) | 20 | La Princesa University Hospital (Madrid) | 7 |
| Donostia University Hospital (San Sebastián) | 20 | Xeral Vigo University Hospital | 7 |
| A Coruña University Hospital | 17 | Rafael Méndez University Hospital | 7 |
| Virgen de la Victoria Hospital (Málaga) | 14 | Morales Meseguer Hospital (Murcia) | 7 |
| Albacete General Hospital | 13 | Virgen de la Concha Hospital (Zamora) | 7 |
| Ntra Señora de la Candelaria University Hospital (Tenerife) | 13 | Naval Viña del Mar Hospital (Valparaíso, Chile) | 6 |
| Dr. Josep Trueta University Hospital (Girona) | 12 | Getafe University Hospital | 5 |
| Virgen de Las Nieves Hospital (Granada) | 12 | Sant Joan University Hospital (Reus) | 5 |
| Ciudad Real General Hospital | 11 | Lozano Blesa University Hospital (Zaragoza) | 5 |
| San Boi General Hospital (Barcelona) | 11 | Clínico University Hospital (Valencia) | 5 |
| Mérida Hospital | 11 | La Fe University Hospital (Valencia) | 5 |
| Puerta de Hierro Hospital (Majadahonda) | 11 | Severo Ochoa University Hospital | 4 |
| Miguel Servet University Hospital (Zaragoza) | 10 | A. Marcide de Ferrol University Hospital | 4 |
| Cruces University Hospital (Barakaldo) | 10 | De los Arcos del Mar Menor Hospital (Murcia) | 3 |
| Vinalopó Hospital (Elche) | 9 | Ntra Señora del Prado Hospital (Talavera) | 3 |
| Río Hortega University Hospital (Valladolid) | 9 | La Mancha Centro Hospital (Alcázar San Juan) | 3 |
| Móstoles University Hospital | 9 | Cáceres University Hospital | 3 |
| Terrassa University Hospital | 9 | Palmaplanas Hospital (Mallorca) | 2 |
| Reina Sofia University Hospital (Murcia) | 8 | Ntra señora de Meritxell Hospital (Andorra) | 2 |
| 12 de Octubre University Hospital (Madrid) | 7 | Burgos University Hospital | 2 |
| La Merced Hospital (Quito, Ecuador) | 7 | Parc de Salut Mar Hospital (Barcelona) | 1 |
| Sagunto Hospital | 7 | Estella Hospital (Navarra) | 1 |
